# Exploring mentalization, trust, communication quality, and alienation in adolescents

**DOI:** 10.1371/journal.pone.0234662

**Published:** 2020-06-15

**Authors:** Angela Clarke, Pamela J. Meredith, Tanya A. Rose

**Affiliations:** 1 School of Health and Rehabilitation Sciences, The University of Queensland, Brisbane, Queensland, Australia; 2 School of Health, Medical and Applied Sciences, Central Queensland University, Rockhampton, Queensland, Australia; Sapienza - University of Roma, ITALY

## Abstract

**Introduction:**

A growing body of evidence has demonstrated the importance of mentalization for adolescents’ psychosocial functioning; however, further research is needed to understand links between mentalization and other socio-cognitive factors. The aim of this quantitative, cross-sectional study was to investigate the relationship between a teen’s capacity to mentalize and three attachment-related factors: parent-teen trust, parent-teen communication, and parent-teen alienation.

**Methods:**

In an online survey, 82 (mainly) Australian adolescents (57 female; 23 male; 2 non-binary; mean age 17.09 years) completed: i) The Children’s Eyes Test, which measured mentalization; and ii) The Inventory of Parent and Peer Attachment-45, which measured trust, communication quality, and alienation.

**Results:**

In teens’ relationships with both mothers and fathers, trust and communication quality were significantly positively correlated (*p* = .001) when controlling for age and gender. Both were significantly negatively correlated with alienation (*p* = .001) with control variables included. Capacity to mentalize did not correlate with trust, communication quality, or alienation in relationships with either mothers or fathers (*p* ≤ .05).

**Conclusions:**

Possible reasons are proposed for why no relationship was found between mentalization and trust, communication quality, or alienation. Implications for future research are discussed.

## Introduction

The period of adolescence is characterized by increased psychosocial opportunities and risks, including heightened vulnerability for the onset of serious mental health conditions such as anxiety and depression [1], psychotic disorders [2], and eating disorders [3]. Researchers have sought to identify factors which might support young people during this period of development, with parent-teen attachment security consistently shown to underpin adolescent well-being [4,5].

In the present study, associations between four attachment-related factors: mentalization, parent-teen trust, parent-teen communication quality, and parent-teen alienation will be explored in adolescents. Adolescence is the transitional phase between childhood and adulthood [6], composed of sub-phases: early adolescence (12 to 14 years); middle adolescence (from 15 to 17 years); and late adolescence (18 to 20 years) [7]. In the following sections, attachment theory, mentalization, trust, communication quality, and alienation will be defined in the context of adolescent development. The importance of these factors to adolescent well-being, as well as known and proposed relationships between these factors will be discussed.

### Attachment theory

Attachment theory is concerned with the nature of the relationship between parents and their children [8]; however, internalized patterns of attachment have been shown to endure [9], with the early parent-child relationship establishing a model for later relationships [10]. The ‘attachment hierarchy’ [11] recognises that individuals can have many attachment relationships, with some more favoured than others. Attachment relationships provide security through the inter-related mechanisms of ‘proximity seeking’, ‘safe haven’, and ‘secure base’ [12]. Specifically, a distressed child will seek proximity to an attachment figure who acts as a safe haven, providing comfort and protection. After the distress subsides, the child can return to exploring the world, assured that the caregiver will continue to be a secure base [13]. Proximity seeking to a secure base continues in adolescence, although with important differences [14] reflecting changes in adolescents’ hierarchy of attachment relationships.

During adolescence, the attachment hierarchy is restructured, with scholars highlighting the increased importance of relationships outside the family [11,15–20]. This change in attachment hierarchy is partially explained by the substantial cognitive and social development occurring during adolescence [14]. First, commencement of formal operational reasoning enables adolescents to think in abstract terms, permitting a re-consideration of existing attachment relationships with caregivers [21]. Second, development occurs within emotional regulation skills, so adolescents may have less need of parents to provide a safe haven [22]. Relatedly, as adolescents’ social networks enlarge [23], parents may be less proximal, prompting adolescents to seek support from peers. Last, adolescents typically desire greater independence from parents [24] as part of identity development [25,26], and may be less willing to accept help from parents [27]. When considering the transfer of the attachment-related functions during adolescence, it is important to acknowledge, however, that relationships with peers do not entirely replace relationships with parents; rather, numerous studies have concluded that parental and peer attachments are positively correlated [28], parent and peer attachments may fulfil different functions in adolescent development [29,30], and parents remain important sources of support for adolescents, particularly in times of danger and distress [31].

Emotionally responsive, consistent care in early childhood fosters a secure internalized attachment pattern (termed ‘autonomous’ in adulthood). If early caregiving is unresponsive or inconsistent, young children can become insecurely attached. Reflecting the work of Ainsworth et al. [32], three categories of insecure attachment have been discussed in the literature: ‘ambivalent/resistant’ (‘anxious/preoccupied’ in adulthood); ‘avoidant’; and ‘disorganised’ (‘unresolved’ in adulthood). Conceptualisations of attachment style in childhood and adulthood have primarily employed this categorical approach [33]; however, some researchers have argued that attachment should be conceptualised as dimensional, specifically, ‘anxiety’ and ‘avoidance’, rather than categorical [34–36]. In contrast, conceptualisation and measurement of adolescent attachment has focussed more on the quality of teens’ relationships with important people, rather than categories or dimensions [36,37].

### Adolescent mentalization

‘Mentalization’, the ability to identify thoughts and emotions in one’s self and others [38], is a socio-cognitive skill that first develops within early parent-child attachment relationships [39]. Mentalization is closely related to the concepts of ‘theory of mind’ [40], ‘reflective functioning’ [41], and ‘perspective-taking’ [42]. A small number of empirical studies have investigated associations between the capacity to mentalize and attachment security in adolescents [43–47]. In each of these studies, links were found between mentalization and attachment. To date, however, there has been no research into possible associations between mentalization and the three other attachment-based factors: trust, communication quality, and alienation in a single sample of adolescents. As each of these factors first develop within the parent-child attachment relationship, it may be reasonable to suggest that associations will be identified.

Although mentalization first develops within the attachment relationship [39], research demonstrates that mentalization skills continue to develop during adolescence [48], supported, in part, by structural and functional changes within the adolescent brain [6]. For instance, increased activity in certain brain regions (left temporo-parietal junction and right dorsolateral prefrontal cortex) in adolescents has been associated with increased sensitivity to others’ perspectives: a central component of mentalization [24]. In addition to neurocognitive changes, adolescents experience new socio-cultural challenges which are relevant to advances in adolescent mentalization. For instance, adolescence is characterised by the broadening of social networks [23], requiring teens to take on new roles, such as worker or romantic partner, and adopt new social conventions [49,50]. Mentalization assists young people to navigate these new social challenges by enabling them to understand their own and others' thoughts, feelings, and intentions [51] and to identify and follow social conventions in new situations [52].

The importance of mentalizing to psychosocial functioning and social communication in adolescence has been well recognised. For instance, mentalization has been found to contribute to the acquisition of affect regulation skills [53,54], resilience [55], and the formation of peer [56] and romantic attachments [57]. Further, mentalization has been identified as a mediating factor between psychopathic traits and aggression [58], and between early childhood maltreatment and onset of personality disorders [59], suggesting that capacity to mentalize might be a protective factor in adolescence [60]. Last, impairment in the capacity to mentalize is considered a factor in the social communication difficulties commonly experienced by individuals living with Autism Spectrum Disorder (ASD) [61], attention-deficit/hyperactivity disorder (AD/HD) [62], psychosis [63], and Borderline Personality Disorder (BPD) [44,64].

With the role of mentalization in psychosocial functioning well established, interest in developing treatment programmes to improve individual mentalization skills has emerged [65]. Mentalization-based treatment (MBT) aims to increase one’s capacity to mentalize [38]; however, only a small number of studies have investigated the effectiveness of MBT in adolescents [40,66–68]. In each of these studies, participants who underwent MBT reported decreased BPD symptoms. In addition, study participants demonstrated: reduced rates of deliberate self-harm and depression [68]; greater trust in parents [40]; and improved quality-of-life [67]. Only Bo et al. [40] and Rossouw and Fonagy [68] measured participants’ mentalizing skills, pre- and post-intervention; in both studies, participants reported better mentalizing skills post-intervention. Whilst these results are promising, the conclusion from a recent review of the evidence-base for MBT was that there is a need to further “…investigate mentalizing and other potential mechanisms of change in MBT…” [69 p. 10].

### Parent-teen trust

Trust in parents develops during early attachment relationships with caregivers [70,71]. Over numerous interactions in which caregivers make and keep promises, individuals develop an expectation that others will act in trustworthy ways, strengthening their capacity to trust others [72]. Adolescents’ ability to form trusting relationships has been linked with their psychosocial adjustment [73–75], willingness to share information with their parents [76,77], and attainment of autonomy within the parent-teen relationship [78,79]. There is evidence that trust increases between early childhood and early adulthood, making adolescence an important age group for the study of trust [80]. Further, most research into trust, to date, has been carried out with young children and adults, and more research related to trust during adolescence is needed [72].

Trust may be underpinned by mentalization as the capacity to mentalize allows an adolescent to infer others’ motivations and intentions when deciding whether or not to trust someone [81]. Empirical research into the links between mentalization and trust in adolescence [24,82–84] has yielded contrasting findings, and further research is needed. For instance, no association between trust behaviours and a deficit in theory of mind was found in a study involving 171 boys (mean age 12.84 years) [83]. In contrast, van den Bos and colleagues [24,84] reported positive links between trust and mentalization in two adolescent samples. Further, the study by Derks et al. [82] identified positive correlations between mentalization and trust, but not in all participants. In the Derks et al. [82] study, 217 teens (mean age 15.11 years) were divided into two groups based on whether they identified as ‘pro-social’ or ‘pro-self’. Both groups completed trust and mentalization measures. No correlation between mentalization and trust was found for pro-social participants; however, in pro-self participants, mentalization was negatively correlated with trust behaviour. In a similar study with young adults (mean age 24.6 years), pro-socials were more reliant on mentalization skills to solve a trust game compared to pro-self participants [85]. Last, brain regions linked to mentalization became activated during trust games in two fMRI studies involving: male and female participants (mean age 21.8 years) [86]; and male participants (mean age 23.6 years) [81].

Importantly, studies have varied in the methods used to measure mentalization and trust, potentially explaining some inconsistency in findings. For example, some studies [24,81,85,86] have utilised functional magnetic resonance imaging (fMRI) to measure mentalizing, with evidence that brain regions important for mentalizing become activated as participants make trust-based decisions. Other researchers [82,83] have used mentalizing tasks such as the Reading the Mind in the Eyes Test (RMET) [87] or the child version, the Children’s Eyes Test (CET) [88]. In the RMET and CET, participants are asked to identify mental states (thoughts or feelings) shown in photographs of the eye region. Studies investigating trust and mentalizing, where mentalizing has been measured using the RMET or CET, have produced contradictory findings. For example, Sharp et al. [83] found no association between participants’ performance in a trust game and results in the CET [88]; however, Derks et al. [82] identified a negative association between participants’ performance in a trust game and the RMET [87]. Each of the studies discussed in this section used ‘trust games’; however, the type of game (e.g., economic trust game, ‘prisoner’s dilemma’ game, ‘ultimatum’ game), and the conditions under which games were played (e.g., familiar partner versus stranger) differed between studies. This factor may also explain inconsistencies in findings. Given the importance of both mentalizing [60] and trust [73] to adolescent well-being, and the present inconsistency in findings, further research into associations between these two factors is needed.

### Parent-teen communication quality

Communication quality, defined as the extent and quality of communication with others [89], has been linked with both attachment [90] and trust [76]. Studying communication quality during adolescence is propitious given that communication between parents and their teenage children can be more difficult than at earlier stages of child development [91]. Teens and parents typically renegotiate the ‘rules’ of communication [92]: a key part of adolescents’ individuation from parents [26].

Several studies have shown that parent-teen communication quality is linked with adolescent well-being. For example, adolescents who report good communication with parents also report: greater life satisfaction [93,94]; higher self-esteem [95]; and better psychosocial adjustment [96] than adolescents with poor communication with parents. Parent-teen communication quality has been linked with adolescents’ willingness to discuss troubling matters with their parents [91] which, in turn, enables parents to monitor their teen’s behaviour and give advice and support [94]. Other studies have linked poorer parent-teen communication with negative outcomes, including: risky sexual activity [97]; problematic internet usage [7,98–100]; smoking and drinking [101]; suicidality [102]; and eating disorders [103].

To date, no studies have investigated mentalization and communication quality; however, it is possible to draw on research into mentalization and another aspect of communication, specifically, ‘pragmatics’ [61]. Pragmatics includes: understanding the ‘rules’ of conversation [52]; comprehending non-literal language, such as sarcasm [104]; and considering communication within its social context [105]. Cummings [106] provided a summary of empirical literature supporting the proposition that mentalization, or ToM, underpins the pragmatics of communication by enabling an individual to infer a communication partners’ thoughts, emotions, and beliefs. Also, a substantial evidence-base has established the role of pragmatic language deficits in social communication difficulties [52]. As mentalization underpins pragmatic aspects of communication, and pragmatics supports the quality of communication with others, it is reasonable to propose a link between mentalization and teens’ self-report on the quality of their communication with their parents. This proposition, however, requires investigation.

### Parent-teen alienation

Alienation, a feeling of being estranged from others [107], has been defined by dimensions such as powerlessness, meaninglessness, and isolation [108]. Researchers have shown that adolescents who reported higher rates of alienation from parents also experienced more psychological health problems [99,109] than adolescents who reported lower rates of alienation from parents. For example, Tambelli et al. [110] invited 816 Italian adolescents (mean age 15.89 years) to report on their relationship with their parents, with alienation from parents predicting participants’ internalizing problems (anxiety and depression). Further, links have been found between parent-teen alienation and higher rates of delinquent behaviour and psychosocial maladjustment [111].

To date, no identified research has investigated mentalization and alienation. Gaining a better understanding of the relationship between these two constructs is important as both may increase a young person’s risk of developing psychological difficulties in adolescence. Links may also have implications for researchers investigating the effectiveness of MBT in adolescents. For instance, alienation may limit an individuals’ ability to engage with clinicians delivering MBT interventions; or, MBT might decrease adolescents’ alienation, which may, in turn, improve adolescents’ relationships with their parents. At present, these clinical questions have not been considered.

### Trust, communication quality, and alienation

Armsden and Greenberg [112] developed the original Inventory of Parent and Peer Attachment (IPPA), proposing that adolescent attachment was based on three factors: trust, communication quality, and alienation. Many studies involving adolescents have revealed relationships among these three factors [113–117]. For example, in Chen’s [115] study of 314 typically-developing Chinese adolescents (mean age 15.13 years), teens’ reports of trust and communication quality for mothers and fathers were significantly positively correlated. Further, both trust and communication quality were significantly negatively correlated with alienation. This pattern of positive correlation between trust and communication quality, and negative correlation with alienation on the IPPA [112], has also been identified in studies involving children [118] and young adults [119].

In summary, research to date suggests possible links between: i) mentalization and trust, and ii) mentalization and pragmatic language skills (which, in turn, have been linked with communication quality). Further, research has established consistent relationships between trust, communication quality, and alienation. No identified studies have investigated associations between either: i) mentalization and communication quality, or, ii) mentalization and alienation. Additionally, no studies have yet considered all four factors together. Understanding the links between mentalization and: trust, communication quality, and alienation may yield information which enhances the capacity of health professionals to tailor their support of adolescents experiencing psychosocial difficulties, including development and implementation of mentalizing-based interventions.

### Hypotheses

The aim of this study was to investigate relationships between four attachment-related factors: mentalization, parent-teen trust, parent-teen communication quality, and parent-teen alienation. Attachment frameworks provide theoretical support for relationships between these four factors. Three hypotheses were proposed. Although studies exploring trust and mentalization have produced mixed findings [24,82–84], hypothesis one drew on theoretical assumptions [120] to propose that teen mentalization would be positively associated with teen trust in both mothers and fathers. Hypothesis two, derived from known links between mentalization and pragmatic language skills [106], and between pragmatic language and communication quality [52], was that teen mentalization would be positively associated with teen communication quality with both mothers and fathers. Based on previous findings reported in the literature [113–117], hypothesis three was that teen trust would be significantly, positively associated with communication quality, and both trust and communication quality would be significantly, negatively associated with alienation (with both mothers and fathers). Although associations between teen mentalization and teen alienation were investigated, no hypothesis was made as no studies exploring these two variables, together, could be found.

## Materials and methods

This was a cross-sectional quantitative study. Written approval was obtained from The University of Queensland Institutional Human Research Ethics Approval Committee. Approval Number: 2017001282.

### Procedure

Convenience [121] and snowball sampling [122] using social media and word of mouth was used to recruit adolescents to complete an online survey using the SurveyMonkey^©^ platform. The survey was initially piloted with five adolescents aged 16-, 17-, or 18-years (2 male; 2 female; 1 non-binary). When asked, the adolescents indicated that no improvements to the survey were needed; thus, no changes were made. The survey was available on SurveyMonkey^©^ for a three-month period. The only inclusion criteria were: chronological age between 16 and 18 years, and adequate English language proficiency (as determined by participants). The age range of 16- to 18-years represents the senior school cohort (grades 11 and 12) in Australia. The choice of this specific age group was a pragmatic decision to enable consistency in participant sampling within a larger programme of research, of which this study forms part. There were no exclusion criteria. Participants were invited to identify the presence/absence of two commonly diagnosed mental health conditions, AD/HD and ASD [123]. No participants identified with AD/HD or ASD; however, verification was not possible as information was provided anonymously via the online survey.

All participants were required to provide informed consent after reading a description of the study, to commence the survey. Participants were not required to answer items that they did not feel willing or able to answer. Ethical approval to conduct the study without gaining parental consent was granted by the ethics committee of the principal researcher’s university on the basis that: i) participants were adolescents aged 16-, 17-, or 18-years, ii) study measures were considered low risk, and iii) participant responses were provided anonymously to researchers.

### Participants

The total study sample comprised 82 adolescents, mean age 17.09 years (*SD* = 0.84). Participant characteristics are reported in Table 1.

**Table 1:** Summary of participant characteristics.

Demographic	n [%]
Age	n = 82
16 years	25 [30.5]
17 years	25 [30.5]
18 years	32 [39.0]
Gender	n = 82
Female	57 [69.5]
Male	23 [28.0]
Intersex or non-binary	2 [2.5]
Living situation	n = 82
Living with both biological parents	62 [75.6]
Not living with both biological parents	20 [24.4]
Primary language spoken at home	n = 81*
English	80 [97.6]
Language other than English	1 [1.2]
Indigenous identity	n = 81*
Did not identify as Indigenous	77 [93.9]
Identified as Indigenous	4 [4.9]
Socio-economic status (based on postcode)^a^	n = 68*
1^st^ quintile (most disadvantaged)	3 [4.4]
2^nd^ quintile	10 [14.7]
3^rd^ quintile	14 [20.6]
4^th^ quintile	11 [16.2]
5^th^ quintile (most advantaged)	30 [44.1]
Attention-Deficit/Hyperactivity Disorder [AD/HD]	n = 82
Had not been diagnosed with AD/HD	82 [100.0]
Had been diagnosed with AD/HD	0 [0.0]
Autism Spectrum Disorder [ASD]	n = 82
Had not been diagnosed with ASD	82 [100.0]
Had been diagnosed with ASD	0 [0.0]

^a^The Australian Bureau of Statistics (ABS) [124]

*Some participants did not provide this information

### Measures

Section one of the online survey asked participants to provide demographic information as reported in Table 1.

In section two, participants completed The Inventory of Parent and Peer Attachment-45 (IPPA-45) [90] which was derived from The Inventory of Parent and Peer Attachment (IPPA) [112]. The original IPPA [112], along with shortened versions and non-English translations, have been widely used in studies of adolescent attachment [89,113,117,125]. The IPPA-45 [90] is shorter than the original IPPA [112] and was validated in Australia with 1,025 adolescents, aged between 13- and 18-years. Wilkinson and Goh [90] concluded that the IPPA-45 demonstrates sound psychometric properties when used with either boys or girls across the age range of adolescence.

The IPPA-45 [90] yields six subscales: i) trust in mother, ii) trust in father, iii) communication quality with mother, iv) communication quality with father, v) alienation from mother, and vi) alienation from father. Participants responded to 30 statements (five per subscale). An example statement describing communication quality is, ‘*I like to get my mother’s point of view on things I’m concerned about*’. Statements were rated on a five-point Likert scale from 1 = ‘strongly disagree’ to 5 = ‘strongly agree’; hence, possible scores ranged from five to 25 for each subscale. Higher scores indicated greater trust, communication quality, and alienation in relationships.

In section three of the online survey, participants completed the Children’s Eyes Test (CET) [88] based on the Reading the Mind in the Eyes Test (RMET) [87]. The CET [88] has been frequently used in empirical studies to provide a measure of mentalization [41,126]. In the CET [88], participants viewed 28 different pictures of the eye region, and were asked to identify the mental state (thought or feeling) depicted. Four words were displayed (one correct and three distracters) and participants chose the option they believed best described what the person in the picture was thinking or feeling. Participants’ correct responses were summed; hence, possible scores ranged from zero to 28, with higher scores indicating greater capacity to mentalize. Adequate psychometric properties have been reported for this measure [88].

## Results

### Statistical analysis

Data were analysed using the IBM Statistical Package for the Social Sciences (SPSS) version 25. A significance level of p ≤ .05 was used for all determinations. Normality was checked by inspection of Q-Q Plots and was considered adequate for each variable. First, descriptive statistics were obtained for: demographic information, mentalization, trust in mother, trust in father, communication quality with mother, communication quality with father, alienation from mother, and alienation from father. Second, one-way between-groups analyses of variance and independent-samples *t*-tests were used to explore the effect of study variables, age and gender, respectively. Third, Pearson’s correlation analyses were used to explore relationships between the research variables.

### Descriptive statistics

Descriptive statistics for the seven research variables are reported in Table 2. Some participants chose not to complete all measures included in the survey, so, pairwise exclusion of missing data was used during statistical analysis [127]. In the current study, Cronbach alpha coefficients for the IPPA-45 subscales: mother trust, mother communication quality, and mother alienation were: .88, .87, and .83 respectively. Cronbach alpha coefficients for father trust, father communication, and father alienation were: .92, .86, and .81, respectively.

**Table 2:** Descriptive statistics for the children’s eyes test and the inventory of parent and peer attachment-45, N = 82.

Variable	n	M	SD	Possible Range	Min.	Max.
Mentalization	76	19.75	3.29	0–28	6	25
Mother Trust	81	20.47	3.91	5–25	8	25
Father Trust	73	18.59	4.96	5–25	5	25
Mother Communication	81	17.72	4.81	5–25	5	25
Father Communication	73	13.46	4.86	5–25	5	23
Mother Alienation	79	13.76	4.39	5–25	6	25
Father Alienation	69	15.30	4.20	5–25	6	25

### Preliminary analysis

Associations between age and gender and the study variables were investigated to determine the need to control for these in later analyses. One-way between-groups analyses of variance were used to explore the effect of age (16-, 17-, or 18-years) on the study variables. There was a statistically significant difference at the *p* ≤ .05 level for the three age groups in both mother alienation (*F* [2,76] = 3.51, *p* = .04) and father alienation (*F* [2,66] = 3.92, *p* = .03). For mother alienation, the effect size, calculated using eta squared, was .08, which is moderate [128 p. 284–7]. Post-hoc comparisons using the Tukey HSD test indicated that 16-year olds (*M* = 15.29, *SD* = 4.30) were significantly more alienated from their mothers compared to 18-year olds (*M* = 12.31, *SD* = 4.08). For father alienation, the effect size, calculated using eta squared, was .11, which is moderate to large. Post-hoc comparisons using the Tukey HSD test also indicated that 16-year olds (*M* = 16.57, *SD* = 3.70) were significantly more alienated from their fathers compared to 18-year olds (*M* = 13.52, *SD* = 3.33). No statistically significant differences between age groups were found for any of the other study variables: mentalization, trust, or communication quality.

Independent-samples *t*-tests were conducted to compare responses from males and females on the study variables. Results obtained from the two participants who identified as non-binary were not included in this analysis. For father alienation, there was a statistically significant difference at the *p* ≤ .05 level between males (*M* = 13.25, *SD* = 4.33) and females (*M* = 16.04, *SD* = 3.86), with female teens reporting greater alienation from fathers than male teens (*t* [66] = -2.62, *p* = .01). The magnitude of the difference in the means (mean difference = -2.79, 95% C*I*:-4.92 to -.66) was small (eta squared = .01). No statistically significant differences between males and females were found for any of the other study variables: mentalization, trust, communication quality, or alienation from mother. As a result of this preliminary analysis, age and gender were controlled for in later analyses involving both mother and father alienation.

### Associations between research variables: Mentalization, trust, communication quality, and alienation

To address the aims of this study, Pearson’s correlation analyses were used to explore relationships between teens’: mentalization and trust (hypothesis one); mentalization and communication quality (hypothesis two); trust, communication quality, and alienation (hypothesis three); and mentalization and alienation (no hypothesis made). For mother and father alienation, reported *r* values are partial correlations after controlling for age and gender. The results are presented in Table 3.

**Table 3:** Summary of partial correlations for major study variables, N = 82.

Variable	1	2	3	4	5	6
1.Mentalization	-					
2. Mother Trust	.14	-				
3. Father Trust	.05	.53**	-			
4. Mother Communication	.11	.73**	.37**	-		
5. Father Communication	.14	.50**	.73**	.59**	-	
6. Mother Alienation	-.16	-.72**	-.33**	-.82**	-.39**	-
7. Father Alienation	-.13	-.50**	-.72**	-.57**	-.75**	.49**

** *p* ≤ .01, two-tailed

No statistically significant correlations were found between the capacity to mentalize and any of the other variables: trust, communication quality, or alienation, with either mothers or fathers (*p* ≤ .05); thus, no further analyses were conducted in relation to mentalization. In relation to teens’ relationships with both mothers and fathers, trust and communication quality were significantly positively correlated, and both trust and communication quality were significantly negatively correlated with alienation (*p* = < .01) after controlling for age and gender.

## Discussion and conclusion

The aim of this study was to investigate associations between four attachment-related variables: mentalization, parent-teen trust, parent-teen communication quality, and parent-teen alienation. This was the first study to explore all four factors together, in a sample of adolescents aged 16-, 17-, and 18-years. Further, this was the first study to explore associations between: mentalization and communication quality, and mentalization and alienation.

### Preliminary analysis

Preliminary analyses in this study revealed age and gender differences in parent-teen alienation (but not for other study variables). First, 16-year old participants reported significantly more alienation from mothers and fathers, compared to 18-year old participants. Developmental changes occurring across the three sub-phases of adolescence may help explain this result. Whilst early adolescence (12- to 14-years) is defined by puberty-related physical and emotional changes, middle adolescence (15- to 17-years) is typically characterised by teens’ desire for individuation/separation from parents and the formation of new, extra-familial relationships [7]. In middle adolescence, friends become the major source of emotional and social support [36], and middle adolescence can involve increased conflict as parent-teen relationships are renegotiated [129]. It is reasonable to propose that teens’ pursuit of individuation/separation, along with greater conflict may, in turn, lead to increased alienation from parents. In contrast, late adolescence (18- to 20-years) involves identity consolidation [26] and there is evidence for a decline in conflict with parents in this period [129]. Interestingly, Sandhu and Tung [130] found that successful identity development was associated with less alienation in 200 males and females aged 18- to 21-years.

Second, in the present study, female teens reported significantly greater alienation from fathers compared to male teens. Higher rates of parental alienation amongst females, compared to males, has been identified in several studies [7,89,90,131]. For instance, amongst 1059 Italian adolescents (mean age 15.66 years) female participants reported significantly greater alienation from fathers compared to male participants [89]. Similarly, amongst 1,025 Australian teens (mean age 16.79 years) female teens reported significantly more paternal alienation than male teens, although both genders reported significantly more paternal than maternal alienation [90]. Scholars have suggested that female teens may report more parental alienation than male teens, as parents place greater expectations on daughters to fulfil roles within the family [28] and there may be more reluctance to support daughters’ individuation/separation, compared to sons [131].

### Associations between research variables: Mentalization, trust, communication quality, and alienation

#### Mentalization and trust

Results for the first hypothesis were not as anticipated. Specifically, the prediction that adolescents’ scores on the mentalization task would be significantly, positively correlated with adolescents’ trust in parents was not supported by the data. Theoretical assumptions [120,132] have supported a relationship between mentalization and trust, arguing that trust-based decisions rely, in part, on the capacity to understand another’s thoughts, emotions, and intentions. To date, however, empirical evidence for a relationship between trust and mentalization has been inconclusive [24,82–84]. Possible reasons for the lack of support for hypothesis one must now be considered.

The use of the IPPA-45 [90] trust subscale to measure parent-teen trust may be one reason that no relationship was found between trust and mentalization. In the development of the original IPPA, Armsden and Greenberg [112] conceptualised secure parent-teen attachment as characterised by high levels of trust and communication, and low levels of alienation in the parent-teen relationship. Importantly, the IPPA trust subscale was not developed as a measure of teen trust, per se. Future studies may consider the use of alternative measures of participant trust, such as the Relational Ethics Scale [133] or the Children’s Generalized Trust Belief Scale [134].

The use of the Children’s Eyes Test (CET) [88] to measure participants’ capacity to mentalize may also help explain why no relationship was found between mentalization and any of the other factors: trust, communication quality, and alienation. Although the original RMET [87] and the CET [88] are popular measures of mentalization [135], concerns about these tools have been discussed in the literature. For instance, the RMET and CET measure only one aspect of mentalization; that is, emotion recognition in others [135]. The RMET and CET do not tap other domains of mentalization [136], such as mentalizing one’s own mental states or reading others’ intentions [83]. The need for more ecologically valid measures of mentalization has been recognised; that is, measures that are closer to everyday situations [126,137]. In addition, several researchers have reported poor correlations between the RMET and alternative tests of mentalizing [135,138]. On this basis, the hypotheses of this study warrant re-consideration using an alternative measure of mentalizing, such as the Reflective Functioning Questionnaire for Youth (RFQY) [139]. The RFQY has been shown to have adequate reliability and validity when measuring adolescent mentalization [41].

#### Mentalization and communication quality

Results for the second hypothesis were not as anticipated. Specifically, the prediction that adolescents’ scores on the mentalization task would be significantly, positively correlated with the quality of adolescents’ communication in relationships with parents, was not supported by the data. In considering reasons for why no relationship was found, it should be remembered that the IPPA-45 [90] measures teens’ reports of the quality of their communication with their parents: it does not measure pragmatic language skills. A relationship between mentalization and communication quality had been proposed based on known links between mentalization and pragmatics [61,106,140,141] and between pragmatics and communication quality [52]. It may be that mentalization is linked with communication quality, but this relationship is via pragmatic language functioning. As pragmatic language was not measured in the present study, this proposition is yet to be explored. Additionally, mentalization may be linked with communication quality via another aspect of language, such as receptive vocabulary. There is a substantial evidence-base supporting an association between mentalization and receptive vocabulary skills [142]. Future research into proposed links between mentalization and communication quality may benefit from the inclusion of standardized assessments of pragmatic language functioning and receptive vocabulary, in addition to a measure of communication quality.

#### Mentalization and alienation

In the absence of studies considering associations between teen mentalization and alienation, no hypothesis had been made. The data in this study revealed no statistically significant relationship between adolescents’ mentalization and adolescents’ sense of alienation from parents. Similar to the discussion regarding use of the IPPA-45 [90] trust subscale, the IPPA-45 alienation subscale was not developed as a measure of teen alienation, per se. Future studies may consider the use of alternative measures of participant alienation, such as The Alienation Scale [143] or The Inventory of Alienation Toward Parents [144].

#### Trust, communication quality, and alienation

The third hypothesis in this study: that adolescents’ trust would be significantly, positively correlated with communication quality, and both trust and communication quality would be negatively correlated with self-reported alienation, was supported. This finding was consistent with results from numerous previous studies [113–117] which have utilised versions of the IPPA. This association between trust, communication quality, and alienation suggests that, where young people have difficulty in one aspect of the parent-teen relationship, there may be difficulties in other areas of the relationship. Thus, where young people report feelings of alienation from their parents, clinicians may choose to explore the quality of trust and communication within the parent-teen relationship. Conversely, where young people report difficulties with trust or communication, a clinician may also investigate a young person’s sense of alienation in relationships with parents.

### Limitations and future research

There are several limitations to this study which may impact interpretation and limit generalisation of findings. First, the sample size was relatively small (N = 82) and only 23 participants were male. Second, reliance on social media and word-of-mouth for recruitment resulted in selection bias: young people with access to technology and adequate English language skills self-selected into the study, resulting in a sample of participants residing largely in areas of high socio-economic advantage. It may be that associations between mentalization and trust, communication quality, and alienation are less evident in young people from socio-economically advantaged families. Future research can address these concerns by: i) assessing a larger number of young people; ii) including those with poor communication skills and from non-English speaking backgrounds; iii) recruiting both typically-developing adolescents and adolescents living with a mental illness; and, iv) involving participants from a broader range of socio-economic advantage. Third, this study utilised self-report measures of all variables, which may have introduced inflated associations due to social desirability response bias [145] and shared method variance [146]. Inclusion of both self-report and standardized assessment tools should be considered in future research. Fourth, the authors acknowledge that the presence of psychopathologies, such as AD/HD and ASD, may have influenced participants’ responses to study measures. As this was an online study with a convenience sample, the presence or absence of psychopathology in study participants could not be ascertained. Whilst participants were invited to identify the presence of two commonly diagnosed mental health conditions, AD/HD and ASD [123] in Section one of the survey, neither condition was reported in this sample. As no participants in the present sample identified with AD/HD and ASD, and other psychopathology was not measured, future research should screen for the presence of psychopathology in participants. In this way, the knowledge-base about mentalization, trust, communication quality, and alienation in young people experiencing mental illness may be expanded.

Although commonly used measures were utilised in the current study, no associations were found between mentalization and the other research variables. As noted earlier, future studies may include alternative measures of adolescent mentalization, trust, and alienation. Further, future exploration of a link between mentalization and communication quality may benefit from the inclusion of measures of pragmatic language and receptive vocabulary, in addition to a measure of communication quality. Finally, although this study explored associations between four attachment-related variables, no measure of attachment, per se, was included in the analyses. Different versions of The Inventory of Parent and Peer Attachment (IPPA) [112] have been widely used in studies of adolescent attachment [89,113,117,125]; however, the IPPA [112] has been criticised as a tool for measuring attachment as it focusses on the quality of the parent-teen relationship [36,102,147,148], rather than adolescents’ internalised attachment style. A future study may address this concern by including a measure of internalised attachment style in adolescence, such as the Experiences of Close Relationships- Revised-General Short Form [36].

## Conclusion

This study involving adolescents aged between 16- and 18-years revealed that the ability to mentalize, as measured by the CET [88], was not associated with three attachment-related factors: parent-teen trust, communication quality, or alienation, as measured by the IPPA-45 [90]. Despite this lack of significant findings, further investigation of mentalization and other socio-cognitive factors in adolescence is important. The recognition of the importance of mentalization to psychosocial well-being is reflected in the strong interest in MBT programmes for individuals living with mental health disorders. Enhancing knowledge about the interplay between mentalization and other factors may assist clinicians and researchers to design and implement effective treatment programmes to improve mentalizing, leading to better mental health outcomes and stronger parent-teen relationships.
